# Genetic Diversity of Porcine Circovirus Types 2 and 3 in Wild Boar in Italy

**DOI:** 10.3390/ani12080953

**Published:** 2022-04-07

**Authors:** Angela Fanelli, Francesco Pellegrini, Michele Camero, Cristiana Catella, Domenico Buonavoglia, Giovanna Fusco, Vito Martella, Gianvito Lanave

**Affiliations:** 1 Department of Veterinary Medicine, University Aldo Moro of Bari, 70010 Valenzano, BA, Italy; francesco.pellegrini@uniba.it (F.P.); michele.camero@uniba.it (M.C.); cristiana.catella@uniba.it (C.C.); domenico.buonavoglia@uniba.it (D.B.); vito.martella@uniba.it (V.M.); 2 Istituto Zooprofilattico Sperimentale del Mezzogiorno, 80055 Portici, NA, Italy; giovanna.fusco@izsmportici.it

**Keywords:** porcine circoviruses, viral diseases, wild boar, Southern Italy, Circoviridae, swine diseases

## Abstract

**Simple Summary:**

Porcine circoviruses (PCVs) are major viral agents of farmed swine, causing relevant economic impact due to direct losses and control measures expenses. Wild boar may serve as a PCVs reservoir for the domestic pigs, thus threatening for production efficiency in pig farms. PCV infection in both domestic pigs and wild boar has been chiefly studied in Northern Italy, a densely populated area with a highly developed pork industry. However, data on circulation of PCV are scarce in other Italian areas. For the above reasons, along with the increasing sanitary relevance of wildlife as host of many livestock diseases, we carried out an epidemiological study to assess the prevalence and genetic characteristics of the PCVs circulating in wild boar in Basilicata region (Southern Italy), characterized by large forested areas with limited anthropic presence. A high prevalence was detected, suggesting that PCVs infection is endemic in the study population. These findings are of particular interest as the pig production system of the study area involves local breeds raised outdoor for the production of high-quality cured meat, thus having a high risk of being in contact with infected wild boar.

**Abstract:**

Porcine circovirus (PCV) infection is associated with relevant economic impact to the pig industry. To date, four species of PCV (PCV1 to 4) have been identified but only PCV2 has been associated firmly with disease in pigs. The objective of this study was to assess the prevalence of PCV2 and PCV3 in the wild boar population in Basilicata region, Southern Italy, since this region is characterized by large forested and rural areas and the anthropic pressure is lower than in other Italian regions. Liver samples from 82 hunted wild boar were collected in 2021 from 3 different hunting districts. Sixty (73%, 95%CI: 63–82) samples tested positive for PCVs by quantitative PCR. In detail, 22 (27%, 95%CI: 18–37) were positive for PCV2, 58 (71%, 95%CI: 60–79) for PCV3, and 20 (24.4%, 95%CI 16–35) for both PCV2 and PCV3. On genome sequencing, different types and sub-types of PCV2 and PCV3 were identified, remarking a genetic diversity and hinting to a global circulation for the identified PCV strains. Overall, the high prevalence suggests that PCV2 and PCV3 infections are endemic in the wild boar population, posing risks for semi-intensive and free-range pig farming, typical of this region, due to contact with PCV-infected wild boar.

## 1. Introduction

Porcine circoviruses (PCVs) (family Circoviridae, genus *Circovirus*) are the smallest known viruses infecting swine populations [1]. PCVs are non-enveloped single-stranded ambisense DNA viruses with a genome of approximately 2 kb, with two open reading frames (ORFs). ORF1 codes for the replication-associated protein Rep whilst ORF2 for the Cap protein which is the only constituent of the viral capsid and it is responsible for viral attachment, contributes to host and cell tropism, and is the main target of the host immune response. To date, four PCV species have been recognized. PCV type 1 (PCV1), PCV2, PCV3 have been reported consistently in swine populations worldwide [2,3,4], whilst PCV4 has been described in domestic pigs with severe clinical diseases in Asia [4,5] and has not yet been identified in European countries [6]. PCV1 is regarded as non-pathogenic to pigs [7]. Conversely, PCV2 infection is associated with diseases of relevant economic impact to the pig industry due to both direct losses and control measures costs. PCV2 is recognized as the primary causative agent of porcine circovirus diseases (PCVD) including postweaning multisystemic wasting syndrome (PMWS), porcine dermatitis and nephropathy syndrome (PDNS) and is also associated with reproductive disorders, respiratory signs, and myocarditis [1]. PCV3 has been also frequently reported in animals showing several clinical outcomes, despite the definition of its pathogenicity is still controversial [8,9]. PCV-associated disorders are considered of multifactorial nature as the viruses need environmental, managerial, host-related co-factors and possible co-infections to trigger the clinical disease [1,9].

PCVs have been reported to infect other species, i.e., ruminants, rodents, canids, and insects (International Committee on Taxonomy of viruses, ICTV, https://talk.ictvonline.org/ictv-reports/ictv_online_report/ssdna-viruses/w/circoviridae/659/genus-circovirus, accessed on 1 March 2022) with the wild boar (*Sus scrofa scrofa*) regarded as relevant reservoir hosts for these pathogens [10,11,12]. PCV2 and PCV3 have been detected in wild boar populations worldwide with high infection rates [11,12]. PCV2-infected wild boar seems to be able to develop the PMWS [13,14], whereas to date there is no evidence of the disease in PCV3-infected wild boar.

A few studies have investigated the presence of PCV2 and PCV3 in wild boar populations in Italy [12,15,16,17]. These studies were mostly designed in densely populated regions with highly anthropized areas and/or with highly developed intensive pork production. However, there is limited knowledge on PCV infection in wild boar populations in scarcely anthropized areas, where pressure of human activities on the environment is expected to be less impactful, i.e., closer to natural conditions. This would help us understand better if wild boar natively harbors PCVs or rather if their virome reflects exposure to human derived activities, i.e., the impact of pig farming. The Basilicata region is characterized by large forested and rural areas where human activities are limited or absent. However, the pig production system in this area involves local breeds which are mostly raised outdoor for the production of high-quality cured meat [18], thus having a high risk of coming in contact with infected wild boar. This offers a unique model to explore the ecology of PCVs in wild boar. The aim of this study was to assess the prevalence of PCVs in wild boar in Basilicata and to investigate the genetic diversity of PCVs in the surveyed area.

## 2. Materials and Methods

### 2.1. Study Area

**Figure 1:**
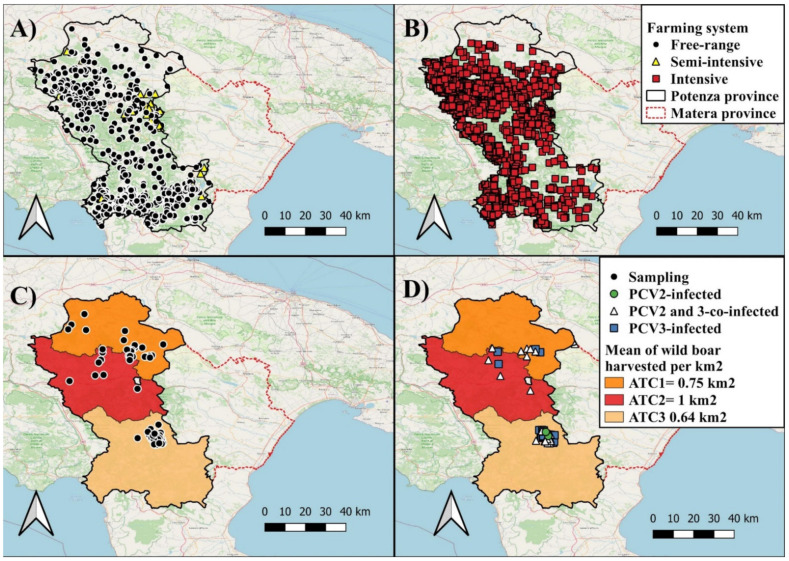
Map of Basilicata region showing distribution of semi-intensive and free-range pig farms (**A**) and intensive pig farms (**B**). Distribution of collected samples (**C**) and of PCV2-positive, PCV3- positive and PCV-2/PCV3-co-infected wild boar in different hunting districts (Ambiti territoriali di Caccia, ATC) (**D**). The choropleth map (**C**,**D**) shows the wild boar density per ATC, computed as the mean of wild boar harvested from 2015/2016 to 2019/2020 hunting season.

The study was carried out in the province of Potenza, Basilicata region (Southern Italy). This area is characterized by large, forested areas [19], representing suitable habitats for the stable presence of wild boar. According to the National Data Base (BDN) for livestock registration, Basilicata region has a swine population of around 67,400 heads and 3030 farms, with 1959 being intensive, 996 free-ranging (outdoor system), and 75 semi-intensive. The majority of the farms (92%) is located in Potenza province, including 1886 intensive farms, 871 free-ranging and 35 semi-intensive (data provided by the Local Competent Authority ASM Basilicata) (Figure 1A,B).

The most appreciated breed for the high quality of its products linked to the local gastronomic tradition is the Suino Nero Lucano characterized by high rusticity and raised in wild or semi-wild conditions [20]. For comparison, the region Lombardia has a swine population of about 4.5 million heads.

In Italy, the National Law 157/92 divided the agro-forestry-pastoral territory of regions into hunting districts named as Ambiti Territoriali di Caccia (ATC). ATCs are of sub-provincial dimensions, possibly homogeneous and delimited by natural borders to be allocated to programmed hunting. Currently, Basilicata is divided into 5 ATCs of which 3 were named as ATC 1, ATC 2 and ATC 3 in the province of Potenza and 2 defined as ATC A and ATC B in the province of Matera.

### 2.2. Sampled Population

Eighty-two liver samples were collected from wild boar during the selective hunting campaign in the 3 different ATCs (20 samples retrieved from ATC 1, 9 from ATC 2 and 53 from ATC 3) from April to July 2021 (Figure 1C). For each animal, information on gender, age, weight, place, and date of hunting were recorded. Subjects were classified as juveniles (<12 months of age), subadults (>12 months and <24 months) and adults (>24 months), based on tooth eruption patterns [21].

### 2.3. Nucleic Acids Extraction

A total of 25 mg of liver tissues were homogenized by a Tissue Lyser (Qiagen GmbH, Hilden, Germany) in 2 mL Eppendorf safe-lock tubes containing 1 mL phosphate-buffered saline solution (PBS) and a 4.8 mm stainless-steel bead (30 Hz for 5 min). Subsequently, homogenates were centrifuged at 10,000× *g* for 3 min. A total of 200 µL of the supernatants were then used for the subsequent nucleic acid extraction using a IndiSpin Pathogen Kit (Indical Bioscience GmbH, Leipzig, Germany), following the manufacturer’s instruction and stored at −80 °C until use.

### 2.4. Molecular Detection of PCV2 and PCV3

The presence of PCV2 and PCV3 DNA was investigated in all the samples by a quantitative real time PCR (qPCR) assay able to simultaneously detect and distinguish between PCV2 and PCV3 [22]. Differential detection was carried out by the use of iTaq™ Universal Probes Supermix (Bio-Rad Laboratories SRL, Segrate, Italy) on a CFX96 Touch™ Real-Time PCR Detection System (Bio-Rad Laboratories SRL, Segrate, Italy). Ten microliters of nucleic acid extract was added to the 15-μL reaction master mix (IQ™ Supermix, Bio-Rad Laboratories Srl) containing 0.6 μM of primers pairs PCV2-F/PCV2-R and PCV3-F/PCV3-R and 0.2 μM of probes PCV2-P and PCV3-P (Table S1). PCV2 and PCV3 copy numbers were calculated on the basis of standard curves generated by 10-fold dilutions of two pEX-A128 standard plasmids containing 300 bp of ORF2 region of PCV2 strain 24657 NL (GenBank accession no. AF201897) and 500 bp of ORF1 region of PCV3 strain Chongqing-147 (GenBank accession no. KY075990), respectively. Both inserted genes were synthetized and cloned by Eurofins Genomics (Ebersberg, Germany). Log10 dilutions of standard DNA were analyzed simultaneously in order to obtain a standard curve for absolute quantification. All standard dilutions and unknown samples were tested in triplicate. Thermal cycling consisted of activation of iTaq DNA polymerase at 95 °C for 3 min and 46 cycles of denaturation at 95 °C for 10 s and annealing extension at 57 °C for 30 s.

Samples tested positive to qPCR screening were subjected to PCR and nested protocols able to amplify short diagnostic genome fragments (<500 nt) of the ORF1 region of PCV2 and PCV3 (Table S1). Primer3 software tool of the Geneious Prime version 2021.2 (Biomatters, Auckland, New Zealand) was used to design PCR and nested PCR primers. Partial ORF1 regions of PCV2 strains representing the current eight (a to h) genotypes known [23] and PCV3 strains representing three genotypes (1, -2 and -3) and relative subtypes (1 for genotype 1, a and b for genotype 2 and a to h for genotype 3) [24] were retrieved from the GenBank database (http://www.ncbi.nlm.nih.gov, accessed on 28 February 2022) and independently aligned to design PCV-2 and PCV-3 species-specific primers. The PCR and nested reactions were performed with a high-fidelity Platinum II Taq Hot-Start DNA Polymerase (Invitrogen, Carlsbard, CA, USA). The thermic file included a first step at 94 °C × 2 min, followed by 35 cycles of 94 °C × 15 s, 55 °C × 15 s and 68 °C × 30 s. One microliter of a 1:100 dilution of the PCR product was used in the nested PCR using the same mix. The thermal protocol was the same as for the first-round PCR. The amplicons with the correct size were purified using specific enzymes (Exo1 and FastaP) or gel purified by a Qiaquick PCR Purification Kit (Qiagen GmbH, Hilden, Germany) to remove primer dimers and/or aspecific bands (incorrect size). The purified PCR products with sufficient DNA concentrations (>10 ng/µL) were subsequently sequenced by Eurofins Genomics (Ebersberg, Germany). Analyses and editing of Sanger sequences were performed using Geneious Prime version 2021.2 (Biomatters Ltd., Auckland, New Zealand) and only high-quality score (>95%) sequences were subjected to further data analysis. Interrogation of the NCBI and EBI sequence databases was performed by the online software tools BLASTN and FASTA Nucleotide in order to retrieve the best hit in the sequence databases.

### 2.5. Strategy for Amplification of Complete Genomes of PCV2 and PCV3

On the basis of the direct sequencing results, samples tested positive for PCV2 and PCV3 with viral load >10^3^ DNA genome copies/mL were selected for full-genome amplification. A rolling circle amplification (RCA) technique [25,26] was performed using the TempliPhi 100 amplification kit (GE Healthcare, Milan, Italy), with minor modifications [27] to increase the number of circular genomes in the samples.

In order to obtain the complete genome of PCV2 and PCV3, inverse PCR and subsequent nested PCR approaches were performed on the RCA products. A set of inverse PCR and nested PCR primers (Table S1) was used to recover the viral circular genome of PCV2 and PCV3 strains, amplifying a fragment of about 2 kb. Primers were designed on the basis of alignments of the partial ORF1 region of PCV2 and PCV3 retrieved from the GenBank database (http://www.ncbi.nlm.nih.gov, accessed on 28 February 2022) using the software Primer 3 plugin of Geneious Prime version 2021.2 (Biomatters Ltd., Auckland, New Zealand). The reverse and forward primers were designed with the 5′ end of the reverse facing the 5′ end of the forward primer. The inverse PCR assay was performed with TaKaRa La Taq polymerase (Takara Bio Europe S.A.S. Saint-Germain-en-Laye, France). Briefly, inverse PCR was performed in a final volume of 50 µL containing 5 µL of RCA product and TaKaRa LA Taq^TM^ (Takara Bio Europe S.A.S., Saint-Germain-en-Laye, France) mix as previously described [27]. The thermal protocol of inverse PCR included a first step at 94 °C × 2 min, followed by 35 cycles of 94 °C for 30 s, 60 °C for 30 s and 68 °C × 3 min, with a final extension of 68 °C × 10 min. One microliter of a 1:100 dilution of the inverse PCR product was used in the nested PCR using the same mix. The thermal protocol was the same as for the inverse PCR. The PCR products were subjected to electrophoresis on a 1.5% agarose gel prepared in TBE buffer (0.09 M of boric acid, 0.09 M of Tris and 0.0025 M of EDTA, pH 8.3) at 50 V for 90 min. PCR amplicons were visualized on a Gel Doc™ EZ (Bio-Rad Laboratories SRL, Segrate, Italy), subjected to purification. Direct Sanger sequencing was performed in both directions by Eurofins Genomics (Ebersberg, Germany).

### 2.6. Sequence and Phylogenetic Analyses

The web-based tools Basic Local Alignment Search Tool (BLAST; http://www.ncbi.nlm.nih.gov, accessed on 28 February 2022) and FASTA (http://www.ebi.ac.uk/fasta33, accessed on 28 February 2022) were employed using the default values to find homologous hits. Sequence editing and multiple codon-based (translation) alignments were performed by Geneious Prime version 2021.2 (Biomatters Ltd., Auckland, New Zealand). The sequences were aligned with cognate PCVs retrieved from the GenBank database by MAFFT [28]. The appropriate substitution model settings for the phylogenetic analysis and estimation of selection pressure on coding sequences were derived using “Find the best protein DNA/Protein Models” implemented in MEGA X version 10.0.5 software [29]. The evolutionary history was inferred by using the maximum-likelihood method, Tamura-Nei 4-parameter model, a discrete gamma distribution and invariant sites to model evolutionary rate differences among sites (6 categories) and supplying statistical support with 1000 replicates. Bayesian inference and neighbor joining methods were also used to explore phylogeny of the strains aligned. The comparison of the phylogenetic trees demonstrated similar topologies with slight differences in bootstrap values at the nodes of the tree. Accordingly, the maximum-likelihood tree was retained.

### 2.7. GenBank Sequence Submission

The nucleotide sequences of strains ITA/2021/351, ITA/2021/382, ITA/2021/397, ITA/2021/413, ITA/2021/415, ITA/2021/330, ITA/2021/369, ITA/2021/371, ITA/2021/386, ITA/2021/432 and ITA/2021/477 employed for phylogeny were deposited in the GenBank database under accession nrs. OM818366- OM818376, respectively.

### 2.8. Statistical Analysis

PCV prevalence was computed along with the 95% confidence interval (95%CI) using the method recommended by Agresti and Coull [30]. The association between PCVs infection and gender was tested using the Chi-square (χ^2^) test. The χ^2^ test for trend was implemented to test for a linear trend over the age categories. Statistical calculations were performed in Epi InfoTM 7.0 [31].

## 3. Results

### 3.1. Epidemiology of PCVs

Out of 82 liver samples collected, 60 (P: 73%, 95%CI: 63–82) animals tested positive for either PCV 2 or PCV3 by qPCR. In particular, 22 (P: 27%, 95%CI: 18–37) were positive for PCV2, 58 (P: 71%, 95%CI: 60–79) for PCV3, and 20 for both PCV2 and PCV3 (P: 24%, 95% 16–35) (Figure 1D) (Table S2). The PCV2 loads ranged from 1.25 × 10^1^ DNA genome copies per mL to 2.49 × 10^5^ DNA genome copies per mL (mean: 2.33 × 10^4^ DNA genome copies/mL, median: 6.48 × 10^2^ DNA genome copies/mL). The PCV3 loads ranged from 1.50 × 10^1^ DNA genome copies per mL to 2.95 × 10^6^ DNA genome copies per mL (mean: 2.47 × 10^5^ DNA genome copies/mL, median: 1.03 × 10^3^ DNA genome copies/mL) (Table S2).

Among the 82 samples collected, the infection rates of PCV in wild boar was 72% (38/53, 95%CI: 58–82) in ATC 3, 75% (15/20, 95%CI: 53–89) in ATC1 and 78% (7/9, 95%CI: 44–95) in ATC 2 (Figure 1D). Out of 22 total PCV2 positive samples identified in the study, 12 (P: 54%, 95%CI: 35–73) were retrieved from livers collected in ATC 3, 6 (P: 27%, 95%CI: 13–48) in ATC 1 and 4 (P: 18%, 95%CI: 7–39) in ATC 2. Out of 58 total PCV3 positive samples, 36 (P: 62%, 95%CI: 49–73) were identified in samples collected in ATC 3, 15 (P: 26%, 95%CI: 16–38) in ATC 1 and 7 (P: 12%, 95%CI: 6–23) in ATC 2 (Table S2).

Out of a total of 60 PCV-positive wild boar, 26 (P: 43%, 95%CI: 31–56) were males and 34 (P: 57%, 95%CI: 44–68) females. In detail, PCV2 was identified in 8 males and 14 females whilst PCV3 was detected in 25 males and 33 females (Table S2). Three percent of PCVs positive animals were juveniles (2/60, 95%CI: 0.2–12), 33% were subadult (20/60, 95%CI: 23–46), and 63% were adults (38/60, 95%CI: 51–74). PCV2 was not detected in juveniles and was identified in 5 subadults and 17 adults, whilst PCV3 was identified in 2 juveniles, 18 subadults and 38 adults (Table S2). No statistically significant differences were detected according to gender and age (*p*-value > 0.05) (Figures S1 and S2).

### 3.2. Sequence Analysis of PCVs

Samples positive for PCV in qPCR were subjected to PCR and nested PCR protocols able to amplify 500 bp-genomic fragments of the ORF1 regions of PCV2 and PCV3. Twenty-seven samples tested positive and were subsequently sequenced, yielding 6 PCV2 and 21 PCV3 sequences (Table S3). Five PCV2 strains identified in this study showed the highest nt identities (99.2–100%) to PCV2 strains retrieved from the GenBank database whilst strain ITA/2021/434 was more distantly related (88.8% nt identity) to strain 71b_Vicenza_36_ (GenBank accession nr KP231135) identified from a domestic pig in Italy in 2008. Out of 21 PCV3 strains, 19 showed the highest nt identities (97.2–100%) to PCV3 strains retrieved from the GenBank database (Table S3) whilst strains ITA/2021/378 and ITA/2021/478 were more distantly related (85.5–86% nt identity) to Chinese strains Nanning2880/2006 (MK814116) and Guizhou-2020 (MZ449237), respectively (Table S3).

The complete PCV genome sequence was obtained only from 11 (5 PCV2 and 6 PCV3) Italian wild boar (Table S4). The genome size of the 5 Italian PCV2 and 6 PCV3 strains identified in this study were 1767 and 2000 nt, like all other PCV2 and PCV3, respectively. The genome features of the identified PCVs comprised two major open reading frames (ORFs), present on complementary strands in the opposite orientation. In the PCV2 strains, the ORF1 (945 nt), located on the virion strand, and the ORF2 (705 nt), located on the opposite strand, encoded for the Rep (315 aa) and Cap (236 aa) proteins, respectively. In the PCV3 strains, the ORF1 (891 nt), located on the virion strand, and the ORF2 (645 nt), located on the complementary strand of the replicative form, encoded for the Rep (297 aa) and Cap (215 aa) proteins, respectively (Table S4). As observed in other PCVs, Italian PCVs contained in the genome two intergenic non-coding regions which were positioned between the start and stop codons of the Rep and Cap protein genes, respectively. The 5′ and 3′ intergenic regions were 34 and 83 nt in length in PCV2 strains and 229 and 235 nt in length in PCV3 strains. The 5′-intergenic regions of PCVs identified in the study encompassed a thermodynamically stable stem-loop, which controls the rolling-circle replication, and the conserved mononucleotide motifs AAGTATTAC in PCV2 strains and TAGTATTAC in PCV3 strains (Table S4).

The nucleotide alignment of the complete genomic sequences of PCV2 and PCV3 strains identified in this report and cognate reference strains recovered in the GenBank database displayed an overall nucleotide (nt) identity ranging from 90.0% to 99.9% among PCV2 strains and 89.6% to 100% among PCV3 strains (Table S5). The Italian PCV2 strains were distinguishable in three different clades. Strains ITA/2021/351, ITA/2021/413, ITA/2021/415 shared the highest nt identity (99.5–99.9%) to PCV2 strain Krasnoyarskiy_2018 (GenBank accession nr MZ511703), which was detected in a domestic pig in Russia in 2018 and classified as subtype 2d [32]. Strain ITA/2021/382 shared the highest nt identity (99.6%) to PCV2 strain serum004 (GenBank accession nr MH287045) identified in a swine in Belgium in 2018 and belonging to subtype 2d-2 [33]. Strain ITA/2021/397 shared the highest nt identity (99.5%) to PCV2 strain V0622 (GenBank accession nr KJ128269) retrieved from a pig in Lithuania in 2009 and classified as subtype 2b [34] (Table S4).

The Italian PCV3 strains were grouped in the same clade. Strain ITA/2021/330 shared the highest nt identity (99.7%) to PCV3 strain SAR1 (GenBank accession nr MN781187) identified in a domestic pig in Sardinia Island, Italy in 2018 [17] of subtype 2a. Strains ITA/2021/369 and ITA/2021/371 shared the highest nt identity (99.6–99.7%) to PCV3 strain HuN-CS (GenBank accession nr MG897478), detected in a pig in China in 2017 and classified as genotype 2a [35]. Strain ITA/2021/386 shared the highest nt identity (99.5%) to PCV3 strain Nanjing 2017 (GenBank accession nr MK580468), identified in a pig in China in 2017, of subtype 2a [36]. Strains ITA/2021/432 and ITA/2021/477 shared 95.6% nt identity each other and the highest nt identity (97.2–97.3%) to PCV3 strain SH11 (GenBank accession nr MN788148), identified in a pig in China in 2018 (Table S4).

**Figure 2:**
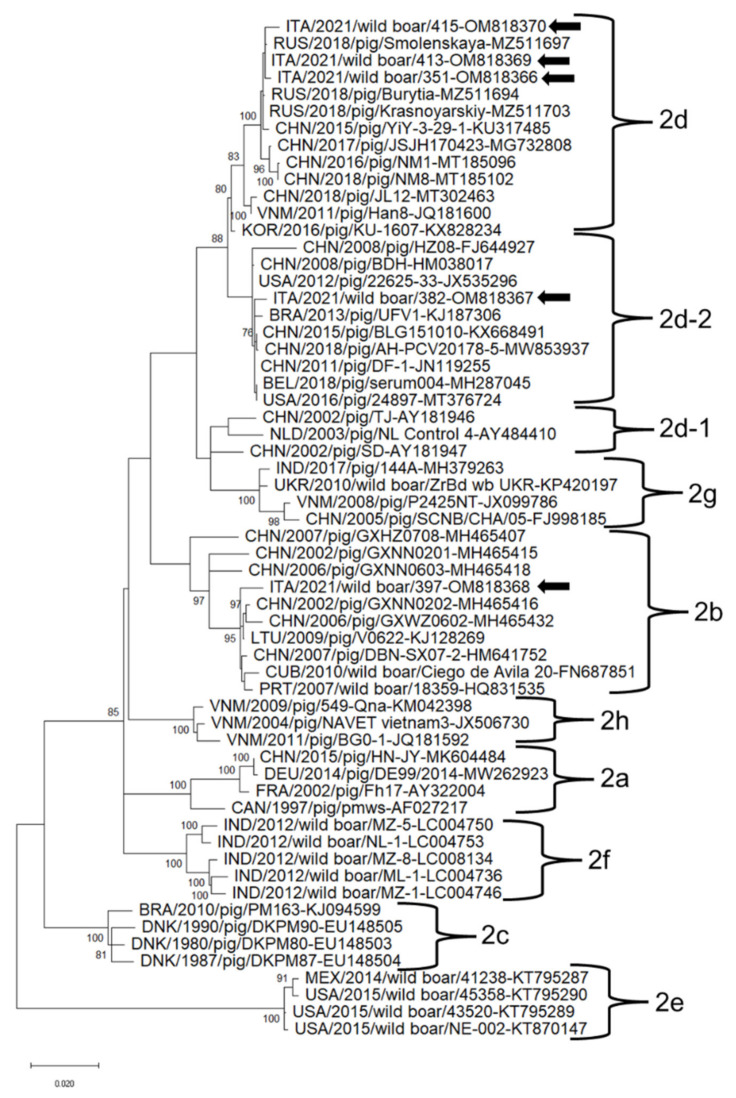
Full-genome-based unrooted phylogenetic tree Porcine circovirus 2 strains identified in this study and reference strains recovered in the GenBank database. The Maximum Likelihood method and Tamura-Nei model (four parameters) with a gamma distribution and invariable sites were used for the phylogeny. A total of 1000 bootstrap replicates were used to estimate the robustness of the individual nodes on the phylogenetic tree. Bootstrap values greater than 70% were indicated. Black arrows indicate strains detected in this study. Numbers of nucleotide substitutions are indicated by the scale bar.

**Figure 3:**
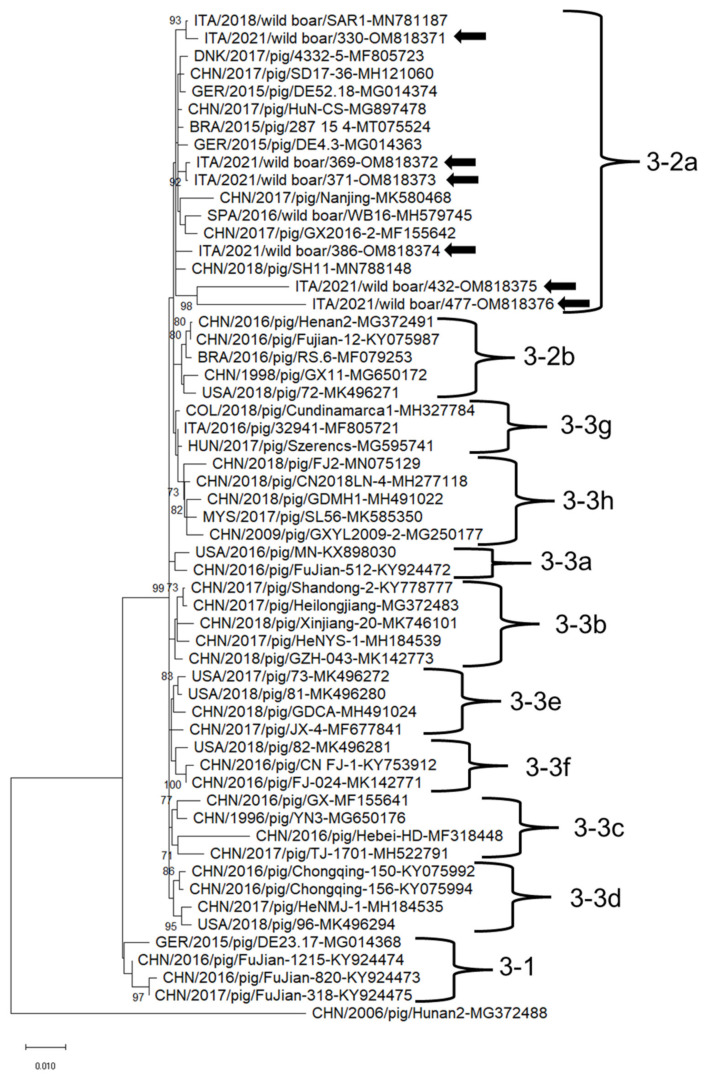
Full-genome-based unrooted phylogenetic tree Porcine circovirus 3 strains identified in this study and reference strains recovered in the GenBank database. The Maximum Likelihood method and Tamura-Nei model (four parameters) with a gamma distribution and invariable sites were used for the phylogeny. A total of 1000 bootstrap replicates were used to estimate the robustness of the individual nodes on the phylogenetic tree. Bootstrap values greater than 70% were indicated. Black arrows indicate strains detected in this study. Numbers of nucleotide substitutions are indicated by the scale bar.

In the phylogenetic tree based on the complete genome nucleotide sequences, the PCV2 and PCV3 strains clustered into different clades together with strains retrieved worldwide (Figure 2 and Figure 3). PCV2 strains ITA/2021/351, ITA/2021/413, ITA/2021/415 belonged to the clade 2d together with Asian and Russian strains (Figure 2). PCV2 strain ITA/2021/382 clustered with other Chinese, American and Belgian strains within clade d-2. PCV2 strain ITA/2021/397 belonged to clade 2b together with Chinese, European and Cuban strains (Figure 3). PCV3 strains ITA/2021/330, ITA/2021/369, ITA/2021/371, ITA/2021/386 clustered within subtype 2a together with other European, Chinese and Brazilian strains. The other two strains ITA/2021/432 and ITA/2021/477 segregated in the clade 2a although they appear more distantly related (Figure 3).

## 4. Discussion

This study reports the circulation of PCV2 and PCV3 in the wild boar population in the Basilicata region. Comparing our data with the literature is difficult since in several studies viral characterization was partial or the studies were focused only a single animal species. In line with a study in wild boar in Campania [15], the cumulative prevalence of PCV2 and PCV3 infection in Basilicata was high (73%), in contrast to the relatively low prevalence reported in domestic pigs in different European countries, Italy included [37]. The infection rate of PCV2 in wild boar in Basilicata (27%) was higher than in Hungary (20.5%) [38], but lower than in other European countries, ranging between 40.3 and 100% [37,39,40,41,42,43,44,45]. Compared with the studies carried out in other parts of Italy, the PCV2 prevalence in wild boar appeared lower than in other regions. PCV2 prevalence was 47.30% in wild boar from Campania region [15], 54.7% in Northern Italy [12] and 81.6% in Sardinia [46]. It should be highlighted that pig production in Sardinia, unlike the rest of the country, is characterized by the abundance of small, non-industrialized pig holdings with low biosecurity and rudimentary infrastructure [47]. Thus, the risk of PCVs spreading within and between domestic pig and wild boar populations should be considered higher in semi-intensive and free-range farming than in intensive pig farming. This hypothesis is apparently weakly supported by the contest and data generated in Basilicata where non intensive farming is largely represented. In our study, most of the PCV-positive samples were retrieved in the hunting district ATC 3 which encompass the highest number (55%, 589/1071) of semi-intensive and free-ranging pig farms of the Basilicata region (Figure 1D). Nevertheless, the prevalence of PCV in our study could have been underestimated due to sampling bias of hunted animals since sampling was not homogeneously distributed across the whole investigated region.

A high prevalence (71%) of PCV3 was observed in the surveyed animals in Basilicata. PCV3 was first discovered in 2016 [9]. PCV3 has been recently described in wild boar in Northern Italy with a prevalence as high as 30% [16]. The prevalence of PCV3 in wild boar in our survey was nearly as high (71%) as in Sardinia Island (77.39% in free ranging pigs and 61.54% in wild boar) [17], but higher than the prevalence of PCV3 detected in the Campania region (49.32%) [15] and in other Mediterranean countries (e.g., 42.66% in Spain) [11].

The high co-infection rate of PCVs (24.4%, 20/82) in wild boar from Basilicata region mirrors the percentage (22.3%) of coinfected animals observed in Campania region [15]. Conversely, the percentage of co-infection of PCV-2 and PCV-3 in pig serum samples from different European countries was lower (3%) [37], thus suggesting an independent circulation pattern of both viruses. 

The data generated in this study could differ from those of previous reports in terms of tissues/organs used for analysis. This could also affect the viral load, as viral replication could be lower in some tissues. Several studies conducted on domestic pigs showed that the detection of the PCV2 and PCV3 genome is easier in tissues than in serum samples [15]. Liver samples from hunted wild boar were used for our screening. Despite other samples (spleen, lung and sera) being also unevenly collected, only liver tissues were available for each of the 82 hunted animals. PCV2 is able to replicate in liver tissue of pigs during natural infection and under experimental conditions [48]. Detection of PCV3 has been reported with high frequency from liver tissues of wild boar, thus suggesting that liver is one of the target organs for PCV-3 replication [11]. Moreover, the average viral load of PCV-2 in serum and tissue samples from domestic swine seems to depend on the stage of infection (PMWS or subclinical infection [15]. Our data showed marked variation in the number of genome copies for both PCV2 and PCV3 in the liver samples. High PCV-2 and PCV-3 viral loads are related to systemic disease, whilst low viral loads are related to subclinical disease (<10^5^ to 10^6^ DNA genome copies/mL in serum) or are retrieved in overtly healthy animals (1.5 × 10^3^ DNA genome copies/mL in the organs) [15]. Accordingly, quantitative information on circovirus DNA could be used as a proxy of PCV2 or PCV3 disease in animals. In all the samples from our collection, the viral load was lower than 10^6^, thus suggesting subclinical or asymptomatic infection.

Noteworthily, no significant difference on PCV infection was observed in terms of age and gender in our survey. Confounding elements in these evaluations are likely present. In some studies, in domestic pigs age-related patterns of PCVs infection have not been identified [49,50]. In other studies, it has been observed that PCV2 can infect pigs from one week of age to adult sows, despite the onset of the disease usually being detected in the weaning group [51]. Moreover, PCV2 detection has been associated with young age of sows in a cross-sectional study [52]. A clear relationship between PCV3 detection rate/load and age (up to 12 months) in wild boar has been also demonstrated [53]. Finally, long-lasting viral infections may occur in wild boar [11]. In another study, gender was identified as a risk factor for PCV infection in wild boar [15].

The increasing number of genome sequences generated for PCV2/PCV3 may pose a challenge for a correct characterization since a consensual classification scheme for types and/or subtypes has not been elaborated. Based on the literature, PCV2 is classified into eight genotypes (from PCV2a to PCV2h) [23], whilst PCV3 has been recently classified into three genotypes (3-1, -2 and -3) and several sub-types [24]. The set of PCV2 strains used as reference along with the strains sequenced in this study showed a 9.9% nt diversity on the basis of a full genome sequence. The Italian strains identified in wild boar clustered with well-recognized clades and were classified as three different genotypes (b, d and d-2). A study in Sardinia Island in Italy has previously evidenced the co-circulation of PCV2b and 2d-2 in pigs and wild-boar [54]. Additionally, in Northern Italy, circulation of PCV2b and 2d strains in domestic pigs has been observed [12]. Interestingly, based on partial replicase sequence, strain ITA/2021/434, shared a low nt identity (88.8%) to other PCV2 strains (Table S3), hinting of a potentially novel type. However, it was not possible to generate the full genome sequence for this strain, hindering a precise genetic characterization.

Based on full genome sequence, all the Italian strains clustered together within clade 2a although two strains were more distantly related. Moreover, on partial sequencing of the replicase, strains ITA/2021/378 and ITA/2021/478 shared a low nt identity (85.5–86.0%) to reference PCV3 sequences retrieved from the GenBank database (Table S3), hinting to the existence of genetically diverse PCV3 strains, yet not described. This would markedly expand the genetic diversity of PCV3. Unfortunately, the full genome sequence of these strains was not obtained.

Overall, these data suggest a marked genetic diversity among the strains retrieved in this study. No geographic clusters could be clearly observed in the PCV2 and PCV3 strains of the surveyed wild boar population. This could be accounted for by phenomena of natal dispersal, and animal migrations [55,56], amplified by the gradual increase in wild boar population size in European countries [57] or even by migrations of hunters [58]. Since we did not survey the local swine population, we do not have information on the genetic diversity of PCV2 and PCV3 in local pig farms and, therefore, making correlations with these data was not possible. However, based on interrogation of the databases, most sequences identified in wild boar in our study matched to circovirus sequences generated from swine worldwide. Additionally, co-segregation of wild boar and swine circovirus sequences within the same clades and sub-clades may suggest a frequent host shift during PCV2 and PCV3 evolution.

## 5. Conclusions

In conclusion, the present study demonstrates widespread circulation of PCVs in the wild boar population in the Basilicata region. The outputs generated by wildlife surveillance, enables us to gain insights that we otherwise would not [59,60,61,62]. In this sense, data from this study provide an important contribution to the literature on PCVs. The high prevalence and the genetic variability of the strains detected suggests that PCV infection is endemic in the study population. The baseline epidemiological data presented herein will be useful for comparative studies. The epidemiological role of wild boar as a potential virus reservoir for domestic pigs, in the light of emerging and re- emerging infectious threats [63], should be monitored with attention.

## Data Availability

Not applicable.

## Supplementary Materials

The following supporting information can be downloaded at: https://www.mdpi.com/article/10.3390/ani12080953/s1, Figure S1: The association between PCVs infection and gender tested using the χ2 test; Figure S2: The χ2 test for trend implemented to test for a linear trend of PCVs infection over the age categories; Table S1: List of oligonucleotides used in this study; Table S2: PCV positive samples by quantitative real-time PCR; Table S3: Interrogation (BLAST) of NCBI nucleotide database (acceesed on 28 February 2022) of the partial (500nt) ORF1 (replicase) sequence of porcine circovirus (PCV) strains generated in this study; Table S4: Genomic features of complete genomes of porcine circoviruses (PCVs) sequenced in this study; Table S5: Nucleotide identity between porcine circovirus 3 (PCV3) strains based on the overall genome.
