# Supplementary material for: Genetic Diversity of Porcine Circovirus Types 2 and 3 in Wild Boar in Italy
Source: Animals (Basel). 2022 Apr 7;12(8):953. doi: 10.3390/ani12080953 (PMC9031215; doi:10.3390/ani12080953)
Supplement: Supplementary file 1 [file animals-12-00953-s001.zip › animals-1658979-supplementary.pdf]

Figure S1: The association between PCVs infection and gender tested using the χ^2 test.

Outcome

	Exposure		Yes	No	Total
FEMALES	Yes		34	11	45
		Row %	75,56%	24,44%	100,00%
		Col %	56,67%	50,00%	54,88%
MALES	No		26	11	37
		Row %	70,27%	29,73%	100,00%
		Col %	43,33%	50,00%	45,12%
	Total		60	22	82
		Row %	73,17%	26,83%	100,00%
		Col %	100,00%	100,00%	100,00%

Odds-based Parameters

	Estimate	Lower	Upper
Odds Ratio	1,3077	0,4911	3,4822
MLE Odds Ratio (Mid-P)	1,3034	0,4803	3,5447
Fisher-Exact		0,4363	3,9054

Statistical Tests

	X²	2 Tailed P
Uncorrected	0,2889	0,59090772
Mantel-Haenszel	0,2854	0,59318001
Corrected	0,0824	0,77404714

Risk-based Parameters

	Estimate	Lower	Upper
Risk Ratio	1,0752	0,8229	1,4049
Risk Difference	5,2853	-14,0687	24,6392

	1 Tailed P	2 Tailed P
Mid-P Exact	0,30052758	
Fisher Exact	0,38590412	0,62420310

Figure S2: The χ^2 test for trend implemented to test for a linear trend of PCVs infection over the age categories

Analysis For Linear Trends In Proportions

Exposure Score	Cases	Controls	Odds Ratio
CLASS 0	2	2	1.000
CLASS 1	20	7	2.857
CLASS 2	38	13	2.923

Add Row

Chi Square for linear trend (Extended Mantel-Haenszel)	0.46467
p value	0.49545

Table S1: List of oligonucleotides used in this study.

Pathogen	Assay	Primers	Sequence (5'-3')	Target gene	Amplification size (bp)	Reference
PCV2/PCV3	qPCR	PCV2-F	CCCTGTCACCCTGGGTGAT	ORF2	77	[20]
		PCV2-R	CCTGTGCCCTTTGAATACTACAGA			
		PCV2-P	FAM-TAAGGTTGAATTCTGGCCCTGCTCCC-Eclipse			
		PCV3-F	CAAGAAAGAGGGGGATTACC	ORF1	69	[20]
		PCV3-R	GCTTGAAGATCCGATCTGG			
		PCV3-P	HEX-GGCGAAGATTCCTC-MGB			
PCV2	PCR	165 F	ACCYCACCTHCAGGGGTTCGCTAA	ORF1	498	this study
		662 R	AGCCAGCCATARAAGTCATCAATA			
	nPCR	176 F	AGGGGTTCGCTAATTTTGTKAARA	ORF1	451	
		626R	TCACCWTGGTAACCATCCCACCACT			
PCV2	inPCR	181 R	STGCTCWGCARCGGTCACCAGACT	ORF1	>1500	this study
		238 F	CGGGAAAATGCAGAAGCGTGAT			
	ninPCR	148 R	YARCAAGGTACTCACAGCAGTAGR			
		307 F	AAGYAAATGGGCTGCTAATTTTGCA			
PCV3	PCR	164 F	ATTTCAAGAACAAAAGGCGACTCA	ORF1	448	this study
		611 R	CCCTCCCCATTATAMCCATCCCAC			
	nPCR	175 F	AAAAGGCGACTCAKCTCGGTGAAG	ORF1	427	
		601 R	TATAMCCATCCCACCAAGGCCC			
PCV3	inPCR	137 R	CGTCTCCGTCAGAATCCGAGC	ORF1	>1500	this study
		193 F	GGCGGGGTTTGCGTGATTTTT			
	ninPCR	69 R	TCTCGAGGTAATCCCCCTCTTTCT			
		296 F	SCGGGAAGCTTGTGMGGATGCKGC			

PCV, porcine circovirus; qPCR, real time quantitative PCR; ORF, Open Reading Frame; nPCR, nested PCR; inPCR, inverse PCR; ninPCR, nested inverse PCR

Table S2: PCV positive samples by quantitative real-time PCR.

N.	Sample code	Gender	Age class	Municipality	hunting district	PCV2 load [a]	PCV3 load [a]
1	330/21	F	2 ***	ARMENTO	ATC 3	-	1.36×10^{5}
2	331/21	M	2	ARMENTO	ATC 3	-	4.88×10^{2}
3	332/21	M	1 **	ARMENTO	ATC 3	-	6.35×10^{4}
4	335/21	F	1	ARMENTO	ATC 3	-	7.88×10^{1}
5	336/21	M	2	ARMENTO	ATC 3	-	9.15×10^{3}
6	347/21	F	1	ARMENTO	ATC 3	-	1.18×10^{4}
7	348/21	F	2	ARMENTO	ATC 3	-	9.75×10^{2}
8	349/21	M	2	ARMENTO	ATC 3	2.37×10^{1}	2.60×10^{3}
9	350/21	M	1	ARMENTO	ATC 3	-	9.18×10^{2}
10	351/21	F	1	ARMENTO	ATC 3	2.38×10^{6}	1.12×10^{4}
11	352/21	F	1	ARMENTO	ATC 3	-	1.78×10^{2}
12	363/21	F	2	POTENZA	ATC 2	-	2.63×10^{4}
13	364/21	M	2	VIETRI	ATC 2	-	$2{,}58 \times 10^{3}$
14	366/21	F	2	ARMENTO	ATC 3	4.88×10^{2}	2.57×10^{3}
15	367/21	F	2	ARMENTO	ATC 3	-	1.19×10^{3}
16	368/21	F	2	ARMENTO	ATC 3	-	1.09×10^{3}
17	369/21	M	1	ARMENTO	ATC 3	-	1.91×10^{5}
18	370/21	M	1	ARMENTO	ATC 3	1.25×10^{1}	-
19	371/21	F	1	ARMENTO	ATC 3	-	9.96×10^{4}
20	372/21	M	1	ARMENTO	ATC 3	-	5.58×10^{2}
21	373/21	F	1	ARMENTO	ATC 3	-	1.91×10^{1}
22	374/21	F	1	ARMENTO	ATC 3	-	5.13×10^{1}
23	376/21	F	2	TRIVIGNO	ATC 2	1.27×10^{1}	3.17×10^{2}
24	377/21	F	2	ARMENTO	ATC 3	-	8.30×10^{2}
25	378/21	F	2	POTENZA	ATC 2	1.23×10^{3}	8.56×10^{3}
26	379/21	M	2	ACERENZA	ATC 1	5.34×10^{3}	7.40×10^{2}
27	381/21	M	2	ACERENZA	ATC 1	-	1.74×10^{1}
28	382/21	F	1	ACERENZA	ATC 1	5.20×10^{3}	7.20×10^{2}
29	383/21	F	1	OPPIDO LUCANO	ATC 3	-	2.34×10^{2}
30	384/21	M	2	ACERENZA	ATC 1	2.55×10^{1}	8.81×10^{2}
31	386/21	M	1	RIONERO IN VULTURE	ATC 1	-	2.49×10^{5}
32	387/21	M	1	ARMENTO	ATC 3	7.37×10^{1}	6.56×10^{2}
33	389/21	M	1	RIONERO IN VULTURE	ATC 1	-	4.50×10^{2}
34	390/21	M	1	ARMENTO	ATC 3	-	1.63×10^{4}
35	391/21	M	1	MELFI	ATC 1	-	5.56×10^{1}
36	394/21	F	2	ARMENTO	ATC 3	5.02×10^{1}	5.06×10^{3}
37	396/21	F	2	ARMENTO	ATC 3	3.69×10^{1}	4.68×10^{2}
38	397/21	F	1	ARMENTO	ATC 3	2.03×10^{3}	-
39	399/21	F	2	ARMENTO	ATC 3	-	1.88×10^{2}
40	400/21	F	2	ARMENTO	ATC 3	-	3.44×10^{4}
41	401/21	M	2	ARMENTO	ATC 3	-	2.76×10^{4}
42	402/21	F	2	MONTEMURRO	ATC 3	3.36×10^{1}	1.86×10^{2}
43	403/21	F	2	ARMENTO	ATC 3	-	6.01×10^{2}
44	406/21	M	2	ARMENTO	ATC 3	-	2.61×10^{2}
45	407/21	F	2	ARMENTO	ATC 3	1.27×10^{1}	2.53×10^{3}
46	408/21	F	2	GUARDIA PERTICARIA	ATC 3	-	9.58×10^{1}
47	410/21	F	2	GENZANO DI LUCANIA	ATC 1	8.08×10^{2}	1.19×10^{4}
48	412/21	M	2	ARMENTO	ATC 3	-	2.61×10^{2}
49	413/21	F	2	ARMENTO	ATC 3	8.42×10^{4}	5.03×10^{3}

50	415/21	M	2	ARMENTO	ATC 3	2.95×10^6	2.53×10^1
51	432/21	M	2	AVGLIANO	ATC 2	2.03×10^1	7.93×10^4
52	434/21	F	2	ACERENZA	ATC 1	3.02×10^3	6.99×10^3
53	436/21	F	2	ACERENZA	ATC 1	-	9.75×10^2
54	459/21	M	2	ACERENZA	ATC 1	2.82×10^3	1.35×10^4
55	476/21	F	2	AVGLIANO	ATC 2	9.30×10^2	5.02×10^4
56	477/21	F	2	AVGLIANO	ATC 2	-	2.07×10^5
57	478/21	M	2	FORENZA	ATC 1	-	4.52×10^3
58	479/21	M	2	FORENZA	ATC 1	-	5.74×10^4
59	499/21-1	F	0 *	OPPIDO LUCANO	ATC 1	-	5.13×10^1
60	499/21-3	M	0	OPPIDO LUCANO	ATC 1	-	1.50×10^1

* Juveniles (<12 months of age); ** Subadults (>12 months and <24 months); *** Adults (>24 months); M, male; F, female; [a] Viral loads are expressed as DNA genome copies/mL; ATC, Ambito Territoriale di Caccia

Table S3: Interrogation (BLAST) of NCBI nucleotide database (February 2022) of the partial (500nt) ORF1 (replicase) sequence of porcine circovirus (PCV) strains generated in this study

N.	Strain	**Identity to reference sequences**		
		PCV species	PCV strain (accession nr.)	nt identity%
1	ITA/2021/330	PCV3	SAR1 (MN781187)	99.5
2	ITA/2021/332	PCV3	SAR1 (MN781187)	99.3
3	ITA/2021/336	PCV3	Sichuan-2020 (MZ449244)	99.8
4	ITA/2021/347	PCV3	WB20GG/2020 (MW287998)	100
5	ITA/2021/351	PCV2	Krasnoyarskiy_2018 (MZ511703)	99.5
6	ITA/2021/363	PCV3	WB20GG/2020 (MW287998)	98.3
7	ITA/2021/364	PCV3	K-2018-1 (MN698819)	98.8
8	ITA/2021/369	PCV3	HuN-CS (MG897478)	99.6
9	ITA/2021/371	PCV3	HuN-CS (MG897478)	99.7
10	ITA/2021/378	PCV3	Nanning2880/2006 (MK814116)	86
11	ITA/2021/382	PCV2	serum004 (MH287045)	99.5
12	ITA/2021/386	PCV3	Nanjing 2017 (MK580468)	100
13	ITA/2021/390	PCV3	HeNYS-2 (MH184540)	97.7
14	ITA/2021/394	PCV3	WB20GG/2020 (MW287998)	100
15	ITA/2021/397	PCV2	V0622 (KJ128269)	100
16	ITA/2021/400	PCV3	WB20GG/2020 (MW287998)	99
17	ITA/2021/401	PCV3	SAR1 (MN781187)	100
18	ITA/2021/410	PCV3	Sichuan-2020 (MZ449244)	99.5
19	ITA/2021/413	PCV2	Krasnoyarskiy_2018 (MZ511703)	99.5
20	ITA/2021/415	PCV2	Krasnoyarskiy_2018 (MZ511703)	99.2
21	ITA/2021/432	PCV3	SH11 (MN788148)	97.3
22	ITA/2021/434	PCV2	71b_Vicenza_36_ (KP231135)	88.8
23	ITA/2021/459	PCV3	1776D/2017 (MG550107)	100
24	ITA/2021/476	PCV3	SW7_5 (MN583565)	99.5
25	ITA/2021/477	PCV3	SH11 (MN788148)	97.2
26	ITA/2021/478	PCV3	Guizhou-2020 (MZ449237)	85.5
27	ITA/2021/479	PCV3	Sichuan-2020 (MZ449244)	100

Table S4: Genomic features of complete genomes of porcine circoviruses (PCVs) sequenced in this study.

Genotype	Sample ID	Accession	Size (nt)	Putative Rep		Putative Cap		5' intergenic region (nt)	3' intergenic region (nt)	Loop motif (5'-3')	Identity to reference sequences		Subtype
				nt	aa	nt	aa				PCV strain ** (accession nr.)	nt identity %	
PCV2	ITA/2021/351	OM818366	1767	945	315	705	236	34	83	AAGTATTAC	Krasnoyarskiy_2018 (MZ511703)	99.8	d
	ITA/2021/382	OM818367	1767	945	315	705	236	34	83	AAGTATTAC	serum004 (MH287045)	99.6	d-2
	ITA/2021/397	OM818368	1767	945	315	705	236	34	83	AAGTATTAC	V0622 (KJ128269)	99.5	b
	ITA/2021/413	OM818369	1767	945	315	705	236	34	83	AAGTATTAC	Krasnoyarskiy_2018 (MZ511703)	99.9	d
	ITA/2021/415	OM818370	1767	945	315	705	236	34	83	AAGTATTAC	Krasnoyarskiy_2018 (MZ511703)	99.5	d
PCV3	ITA/2021/330	OM818371	2000	891 *	297	645	215	229	235	TAGTATTAC	SAR1 (MN781187)	99.7	2a
	ITA/2021/369	OM818372	2000	891 *	297	645	215	229	235	TAGTATTAC	HuN-CS (MG897478)	99.6	2a
	ITA/2021/371	OM818373	2000	891 *	297	645	215	229	235	TAGTATTAC	HuN-CS (MG897478)	99.7	2a
	ITA/2021/386	OM818374	2000	891 *	297	645	215	229	235	TAGTATTAC	Nanjing 2017 (MK580468)	99.5	2a
	ITA/2021/432	OM818375	2000	891 *	297	645	215	229	235	TAGTATTAC	SH11 (MN788148)	97.3	2a
	ITA/2021/477	OM818376	2000	891 *	297	645	215	229	235	TAGTATTAC	SH11 (MN788148)	97.2	2a

nt, nucleotides; aa, amminoacids; * alternative start codon (GTC); ** genome sequence with the highest identity on interrogation of GenBank database with BLAST

Table S5: Nucleotide identity between porcine circovirus 3 (PCV3) strains based on the overall genome.

	PCV3-1	PCV3-2a	PCV3-2b	PCV3-3a	PCV3-3b	PCV3-3c	PCV3-3d	PCV3-3e	PCV3-3f	PCV3-3g	PCV3-3h	ITA/2021/432	ITA/2021/477	CHN/2006/Hunan2
**PCV3-1**	99.0–99.8	97.7–98.5	97.8–98.4	97.9–98.4	97.7–98.5	97.7–98.4	97.9–98.4	97.9–98.4	97.8–98.4	98.2–98.6	97.7–98.3	95.8–96.3	95.5–95.9	91.9–92.5
**PCV3-2a**	97.7–98.5	98.7–99.7	98.1–98.6	97.8–98.4	97.8–98.4	97.6–98.3	97.8–98.5	97.7–98.3	97.6–98.2	97.5–98.3	97.6–98.2	96.8–97.3	96.6–97.2	91.0–91.6
**PCV3-2b**	97.8–98.4	98.1–98.6	99.4–100	98.2–99.2	98.1–99.0	97.9–98.9	97.8–98.8	97.7–98.8	97.6–98.9	97.6–98.8	97.6–98.7	96.9–97.1	96.6–96.8	91.5–91.7
**PCV3-3a**	97.9–98.4	97.8–98.4	98.2–99.2	99.2–99.4	98.3–99.0	98.1–98.9	98.0–98.8	97.9–98.6	97.8–98.6	97.6–98.5	97.6–98.5	96.9–97.0	96.7–97.8	91.1–91.5
**PCV3-3b**	97.7–98.5	97.8–98.4	98.1–99.0	98.3–99.0	99.3–99.9	98.2–98.9	98.1–98.9	97.9–98.8	97.8–98.8	97.6–98.7	97.6–98.7	96.7–97.1	96.5–96.8	91.3–91.8
**PCV3-3c**	97.7–98.4	97.6–98.3	97.9–98.9	98.1–98.9	98.2–98.9	99.0–99.2	98.2–98.7	97.9–98.8	97.8–98.8	97.6–98.7	97.6–98.7	96.5–96.8	96.3–96.7	90.4–91.3
**PCV3-3d**	97.9–98.4	97.8–98.5	97.8–98.8	98.0–98.8	98.1–98.9	98.2–98.7	99.3–99.8	98.1–99.1	98.0–98.0	97.9–98.8	97.9–98.7	96.7–97.0	96.5–96.8	91.3–91.7
**PCV3-3e**	97.9–98.4	97.7–98.3	97.7–98.8	97.9–98.6	97.9–98.8	97.9–98.8	98.1–99.1	99.4–99.7	98.2–99.0	98.1–98.8	97.8–98.7	97.0–97.1	96.7–96.9	91.3–91.4
**PCV3-3f**	97.8–98.4	97.6–98.2	97.6–98.9	97.8–98.6	97.8–98.8	97.8–98.8	98.0–98.0	98.2–99.0	99.3–99.8	98.3–99.1	98.2–98.9	96.8–97.0	96.5–96.7	91.1–91.5
**PCV3-3g**	98.2–98.6	97.5–98.3	97.6–98.8	97.6–98.5	97.6–98.7	97.6–98.7	97.9–98.8	98.1–98.8	98.3–99.1	99.6–99.8	98.2–99.2	97.0–97.1	96.8–96.9	91.4–91.5
**PCV3-3h**	97.7–98.3	97.6–98.2	97.6–98.7	97.6–98.5	97.6–98.7	97.6–98.7	97.9–98.7	97.8–98.7	98.2–98.9	98.2–99.2	99.0–99.6	96.7–97.0	96.5–96.8	91.1–91.3
**ITA/2021/432**	95.8–96.3	96.8–97.3	96.9–97.1	96.9–97.0	96.7–97.1	96.5–96.8	96.7–97.0	97.0–97.1	96.8–97.0	97.0–97.1	96.7–97.0	-	95.6	89.6
**ITA/2021/477**	95.5–95.9	96.6–97.2	96.6–96.8	96.7–97.8	96.5–96.8	96.3–96.7	96.5–96.8	96.7–96.9	96.5–96.7	96.8–96.9	96.5–96.8	95.6	-	89.6
**CHN/2006/Hunan2**	91.9–92.5	91.0–91.6	91.5–91.7	91.1–91.5	91.3–91.8	90.4–91.3	91.3–91.7	91.3–91.4	91.1–91.5	91.4–91.5	91.1–91.3	89.6	89.6	-
